# Donor Small-Droplet Macrovesicular Steatosis Affects Liver Transplant Outcome in HCV-Negative Recipients

**DOI:** 10.1155/2019/5862985

**Published:** 2019-05-02

**Authors:** Flaminia Ferri, Quirino Lai, Antonio Molinaro, Edoardo Poli, Lucia Parlati, Barbara Lattanzi, Gianluca Mennini, Fabio Melandro, Francesco Pugliese, Federica Maldarelli, Alessandro Corsi, Mara Riminucci, Manuela Merli, Massimo Rossi, Stefano Ginanni Corradini

**Affiliations:** ^1^Department of Translational and Precision Medicine, Sapienza University of Rome, 00185 Rome, Italy; ^2^Hepato-Bilio-Pancreatic and Liver Transplant Unit Department of Surgery, Sapienza University of Rome, 00161 Rome, Italy; ^3^Department of Molecular and Clinical Medicine, University of Gothenburg, and Sahlgrenska University Hospital, 405 30 Gothenburg, Sweden; ^4^Centre Hepato-Biliaire, Hôpital Paul Brousse, AP-HP, 94800 Villejuif, France; ^5^Hepatology Department, Université Paris Descartes, Cochin Hospital, AP-HP, 75014 Paris, France; ^6^Department of Anaesthesiology Critical Care Medicine and Pain Therapy, Sapienza University of Rome, 00161 Rome, Italy; ^7^Department of Molecular Medicine, Sapienza University of Rome, 00161 Rome, Italy

## Abstract

**Background:**

No data are available on liver transplantation (LT) outcome and donor liver steatosis, classified as large droplet macrovesicular (Ld-MaS), small-droplet macrovesicular (Sd-MaS), and true microvesicular (MiS), taking into account the recipient Hepatitis C virus (HCV) status.

**Aim:**

We investigate the impact of allograft steatosis reclassified according to the Brunt classification on early graft function and survival after LT.

**Methods:**

We retrospectively reviewed 204 consecutive preischemia biopsies of grafts transplanted in our center during the period 2001-2011 according to recipient HCV status.

**Results:**

The median follow-up after LT was 7.5 years (range: 0.0-16.7). In negative recipients (n=122), graft loss was independently associated with graft Sd-MaS, in multivariable Cox regression models comprehending only pre-/intraoperative variables (HR=1.03, 95%CI=1.01-1.05;* P*=0.003) and when including indexes of early postoperative graft function (HR=1.04, 95%CI=1.02-1.06;* P*=0.001). Graft Sd-MaS>15% showed a risk for graft loss > 2.5-folds in both the models. Graft Sd-MaS>15% was associated with reduced graft ATP content and, only in HCV- recipients, with higher early post-LT serum AST peaks.

**Conclusions:**

In HCV-negative recipients, allografts with >15% Sd-MaS have significantly reduced graft survival and show low ATP and higher AST peaks in the immediate posttransplant period. Donors with >15% Sd-MaS have significantly higher BMI, longer ICU stays, and lower PaO2.

## 1. Introduction

The frequency of steatosis in donors for liver transplantation (LT) is increasing over time, showing similar trends as the general population. Most studies exploring the effect of steatosis on LT outcomes classify it as “macrovesicular” or “microvesicular” steatosis [1]. Based on these studies, it is generally accepted that grafts with severe macrovesicular steatosis (≥60%) should be discarded due to elevated risk of graft failure [2–4]. Although some reports have associated microvesicular steatosis with initial poor graft function, it has been generally accepted that this condition is not associated with reduced graft survival irrespective of the percentage of hepatocytes involved [3, 5–7]. However, most of these studies did not perform routine protocol biopsies in all the donors, consequently selecting a subclass of grafts according to their gross appearance or donor characteristics. Moreover, great inhomogeneity has been reported on the timing of graft biopsies; for example, biopsies performed after donor ischemia may report artifacts in steatosis estimation caused by the development of ischemia-reperfusion damage, such as hepatocellular vacuolization [8]. Interestingly, two studies, in which biopsy was systematically performed in the donor before organ perfusion, reported a poor graft survival using liver grafts with moderate or severe microvesicular steatosis [9, 10].

Poor clarity exists also on a clear histological definition of macro- and microvesicular steatosis. In this respect, an accurate classification of hepatic steatosis has been proposed by Brunt, classifying the steatosis as (a) large droplet macrovesicular (Ld-MaS), (b) small-droplet macrovesicular (Sd-MaS), and (c) true microvesicular steatosis [11]. In the past, Sd-MaS and true microvesicular steatosis, a condition observed in very peculiar conditions like Reye syndrome, drug toxicity, or acute fatty liver of pregnancy, may have been considered as one entity [11]. Although Brunt's classification has been positively accepted in the LT field [12–14], only one study adopted it with the intent to investigate post-LT outcomes. Interestingly, this study reported that allograft Sd-MaS was associated with acute and chronic rejection [15].

Another underestimated aspect to consider is the potential confounding role of HCV infection when we analyze the association between allograft steatosis and post-LT including (a) HCV interaction with lipid metabolism in the hepatocytes [16]; (b) independent association of steatosis with hepatic inflammation and fibrosis in HCV-positive patients [17]; and (c) negative impact of donor macrovesicular steatosis ≥15-30% in HCV-positive recipients [18, 19].

The principal aim of the study was to investigate the impact of allograft steatosis, reclassified according to the Brunt classification, on early graft function and survival after LT. Separate analyses were done in HCV-negative (HCV-) and HCV-positive (HCV+) patients. The secondary aim was to evaluate the ATP levels in the donor livers according to the type and percentages of steatosis.

## 2. Patients and Methods

### 2.1. Patients

During the study period (February 27th, 2001-July 28th, 2011), 233 consecutive adult (≥18 years) patients received a first, nonurgent, deceased-donor, whole-organ LT at Sapienza University of Rome Liver Transplant Center, Italy. Protocol preperfusion donor liver biopsies were prospectively collected for all patients and retrospectively evaluated for reestimating the donor steatosis according to the Brunt classification.

**Figure 1:**
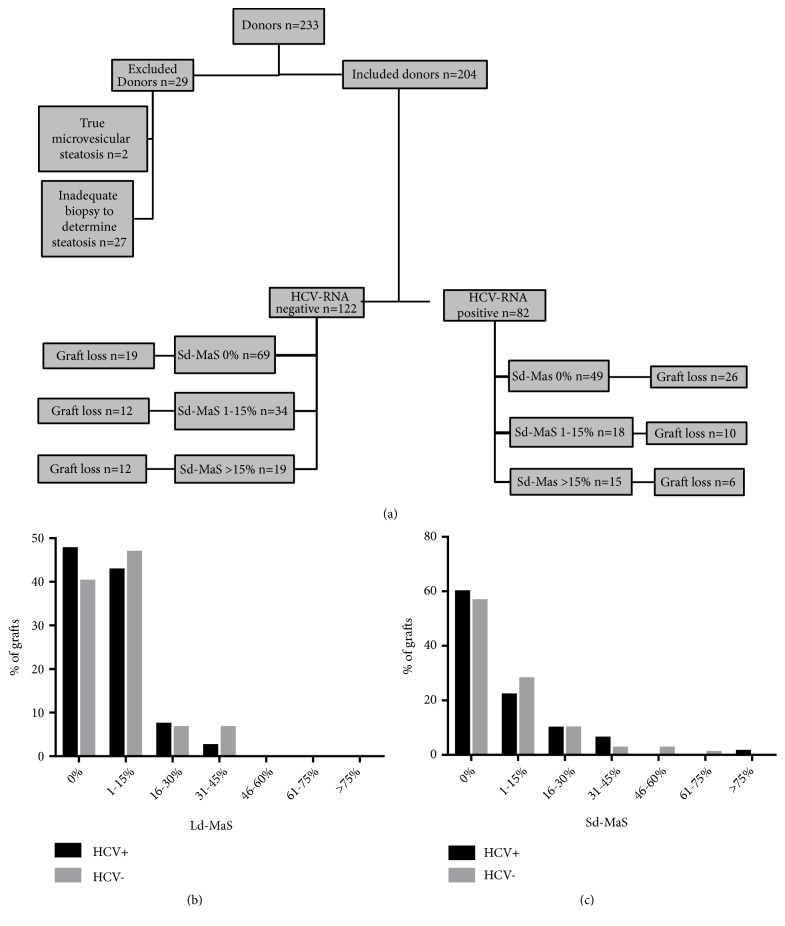
Study population. (a) Flow chart of liver graft loss according to HCV status and Sd-MaS percentage. (b) Graft Ld-MaS distribution in the HCV- and HCV+ groups. (c) Graft Sd-MaS distribution in the HCV- and HCV+ groups.

As shown in Figure 1, exclusion criteria were defined as the exclusive presence of true microvesicular steatosis (n=2) and biopsy inadequate for steatosis evaluation (n=27). The study was conducted on the remaining 204 patients (87.6%). No donor was HCV-Ab positive.

### 2.2. Transplant Aspects

All transplants were performed with a terminoterminal choledochocholedochostomy with T-tube placement. The immunosuppressive protocol was based on a triple therapy with methylprednisolone, mycophenolate mofetil, and calcineurin inhibitor (cyclosporine=40 patients; tacrolimus=166 patients). Methylprednisolone was rapidly tapered. Donor and recipient data were prospectively collected using an in-house database and retrospectively reviewed; donor information was supplemented by data held in the National Transplant Center database. Initial Poor Graft Function (IPGF) was defined according to Nanashima et al. [20]. Early allograft dysfunction (EAD) was defined according to Olthoff et al. [21]. Causes of graft loss were reported and classified as liver-related or liver-unrelated according to the European Liver Transplant Registry [22].

### 2.3. Liver Biopsies

Permanent histological sections were prospectively collected from allograft preischemia liver wedge biopsies performed on the left hepatic lobe. The liver tissue was immediately fixed in 10% formalin and within few days was embedded in paraffin and then stained with hematoxylin and eosin, to assess hepatic steatosis in all transplants. To grade the severity of ischemia-reperfusion injury (IRI), permanent histological sections in the recipient within 1 hour after complete revascularization of the allograft (postreperfusion biopsy) were obtained in 134 cases (79 HCV- and 55 HCV+ patients, respectively) with the same procedure.

**Figure 2:**
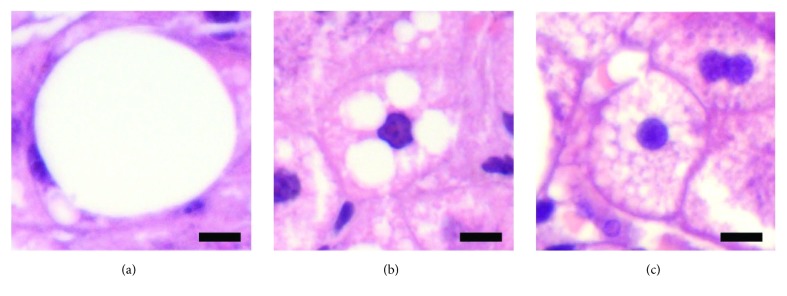
Representative images of Ld-MaS (a), Sd-MaS (b), and true microvesicular steatosis (c). In (a), a single fat vacuole displaced the nucleus to periphery of the cell. In contrast, multiple fat vacuoles not displacing the nucleus were considered the hallmark of (b). In (c) steatosis was true microvesicular when many tiny lipid vesicles were diffusely distributed within the cytoplasm leading to a foamy appearance.

Frozen-section evaluation was performed in selected cases based on gross appearance of the graft only to decide whether to discard the graft. Two expert pathologists (AC and MR), blinded to clinical data and to the frozen-section evaluation, retrospectively reviewed and scored all the preischemia liver samples for steatosis, defined according to the Brunt classification [11, 14] (Figure 2) as follows: Ld-MaS, as one or few large vacuoles in the cytoplasm with eccentric nuclear displacement; Sd-MaS, as few and discrete fat vacuoles that were smaller than half of the cell and did not displace the nucleus. True microvesicular steatosis was defined as the presence of innumerable tiny indiscernible lipid vesicles diffusely distributed in the cytoplasm causing its foamy appearance. Only 2 grafts had true microvesicular steatosis and, as mentioned above, were removed from further analyses. Ld-MaS and Sd-MaS were expressed as percentages of hepatocytes involved. Postreperfusion histopathological IRI score was assessed according to a modified method derived from Suzuki et al. [8]. Briefly, hepatocellular necrosis, sinusoidal congestion, and polymorphonuclear cell infiltration were taken into account. Necrosis was scored as absent [0] or involving single cell [1], less than 30% of hepatocytes [2], 30-59% of hepatocytes [3], and more than 60% of hepatocytes [4]. Sinusoidal congestion was scored as absent [0], minimal [1], mild [2], moderate [3], and severe [4]. Polymorphonuclear cell infiltration was scored according to the number of foci/field as follows: absent [0], ≤ 1 [1], 2-4 [2], 5-10 [3], and > 10 [4]. The ATP graft content was measured in preischemia and postreperfusion biopsies by bioluminescence assay (Molecular Probes® kit).

### 2.4. Statistical Analyses

Continuous variables are presented as median and interquartile ranges (IQR). After assessment of normality by the Kolmogorov-Smirnov test, the differences between groups were evaluated by Mann-Whitney* U* test or T test according to the variable normality. Categorical variables were expressed as count and percentages and compared by the chi-square or Fisher's exact test, as appropriate. As a value over 15% of graft Sd-MaS turned out to be relevant for graft survival in HCV- patients, we decided to categorize both Sd-MaS and Ld-MaS as nil steatosis (absence of steatosis), 1 to 15%, and >15% steatosis. We categorized the total histopathological IRI score in mild/moderate (value <6) and severe (value ≥7), the latter corresponding to the higher tertile in our population.

All the analyses were performed separately in patients with HCV- and HCV+ liver disease.

Survival rates were calculated using the Kaplan-Meier method. In order to calculate graft survival, patients alive and not retransplanted were censored at the date of last follow-up, while time to graft loss was measured from LT to patient death or retransplantation. Patient-, donor-, graft-, and transplant-specific risk factors for overall graft survival were investigated using univariable Cox regression analyses. Different multivariable Cox regression models were constructed, considering as covariates only pre-/intraoperative variables or both pre-/intraoperative and early postoperative variables. Hazard ratios (HR) and 95% confidence intervals (95%CI) were reported.

To investigate donor factors independently associated with graft Sd-MaS >15% compared to a lower degree of Sd-MaS or nil Sd-MaS, we used logistic binary regression.

Variables with a* P* value <0.05 at univariate analyses were introduced as covariates in all the multivariable analyses.

A* P* value <0.05 was considered statistically significant. Computations were carried out with SPSS software 24.0 for Windows (SPSS Inc., Chicago, IL). The study was approved by the Sapienza University of Rome Ethical Committee and patients signed written informed consent forms.

## 3. Results

### 3.1. Recipient, Donor, Graft, Intraoperative, and Early Postoperative Characteristics

The recipient, donor, graft, intraoperative, and early postoperative characteristics of the entire study population are shown in Table 1. All patients with HCV+ liver disease (n=82; 40.2%) were serum HCV-RNA positive at transplant; 32 patients had also HCC which was the only indication to LT in 12 patients. During the post-LT follow-up period, 40 patients achieved a sustained virological response, 20 with Direct-Acting Antivirals (DAAs), and 20 with Pegylated Interferon alpha and Ribavarin. Among the 122 HCV- patients, alcohol-related cirrhosis was the main cause of liver disease (n=36; 29.5%) followed by HBV (n=26; 21.3%) cryptogenic/NASH (n=19; 15.6%), cholestatic disease (n=8; 6.6%), mixed etiologies (n=17; 13.9%), and other causes (n=16; 13.1%); 46 patients had also HCC which was the only indication to LT in 18 patients. No HCV- patient had a previous HCV-RNA positivity. Median donor age was 50.5 years, with 65 (31.9%) cases older than 60 years. Preischemia liver Ld-MaS and Sd-MaS involving >15% of hepatocytes were present in 24 (11.8%) and 34 (16.7%) cases, respectively. Ld-MaS >30% was present in only 10 (4.9%) grafts, with a maximum Ld-MaS value of 40% observed in 6 (2.9%) cases. Sd-MaS ≥40% was present in 9 (4.4%) grafts, with a maximum Sd-MaS value of 80% observed in 1 (0.5%) graft. Figure 1 details graft steatosis distribution in the HCV- and HCV+ groups. An excellent interanalytical correlation (interclass correlation coefficient >0.9) was reported between the two histopathologists concerning steatosis and IRI assessment.

In the entire study population, median follow-up was 7.5 years (range: 0.0-16.7). Comparing HCV- versus HCV+ patients, the only difference was that Anti-HBc positive donors were less frequently allocated to HCV+ patients (*P*=0.043).

### 3.2. Variables Associated with Graft Survival in HCV-Negative Patients

In the HCV- group, the median follow-up was 7.8 years (range: 0.0-16.7). During the follow-up period, 28 grafts (22.9%) were lost for liver-related causes. In detail, we observed nine cases of delayed graft dysfunctions, six ischemic cholangitides, four primary nonfunctions, three HCC recurrences, two hepatic artery thromboses, one acute rejection, one chronic rejection, one recurrence of primary biliary cholangitis, and one portal thrombosis. Fifteen (12.3%) liver-unrelated causes for graft loss were observed (six de novo malignancies, three cerebrovascular accidents, three acute myocardial infarctions, two cases of sepsis, and one multiorgan failure). One-, 3-, and 5-year graft survival rates were 82.8%, 76.2%, and 71.3%, respectively. As shown in Figure 1, twelve out of nineteen grafts with Sd-MaS >15% were lost. At univariable Cox regression (Table 2), graft Sd-MaS was a risk factor for graft loss, when considered as a continuous (*P*<0.001) or a categorized (>15%) variable (*P*=0.002). Other risk factors for graft loss were donor Anti-HBc positivity (*P*=0.001), longer time since transplantation (*P*=0.036), and the occurrence of IPGF (*P*=0.025) and EAD (*P*=0.008). On the opposite, Graft Ld-MaS, IRI severity, HCC, and other studied variables were not associated with overall graft survival (Table 2).

Two multivariable Cox regression models were created (Table 3), the first including only pre-/intraoperative significant variables and the second including postoperative significant ones. In both models, only Anti-HBc positivity and Sd-MaS >15% were independent risk factors for graft loss (*P*=0.001 in the pre-/intraoperative model and* P*=0.007 in postoperative model). Grafts with Sd-MaS >15% had a 2.5-fold increased risk for graft loss in both the models (*P*=0.008 in the pre-/intraoperative model and* P*=0.019 in postoperative model). When Sd-MaS was considered as a continuous variable, it was an independent risk factor for graft loss with HRs 1.036 (*P*=0.001) and 1.032 (*P*=0.003) in the pre-/intraoperative model and in that including postoperative variables, respectively.

Although not significant in the univariate model, we decided to test the Ld-MaS in separate analyses, with the main intent to exclude a possible effect of coexisting Sd-MaS and Ld-MaS on graft loss. After having constructed the same multivariable models based on pre-/intraoperative and pre-/intra-/postoperative variables plus the variable Ld-MaS, Sd-MaS >15%, we confirmed its independent role of Sd-MaS >15% as a risk factor for graft loss, with HRs 3.311 (*P*=0.015) and 3.157 (*P*=0.021) in the two models, respectively. Ld-MaS was not significant in these models.

**Figure 3:**
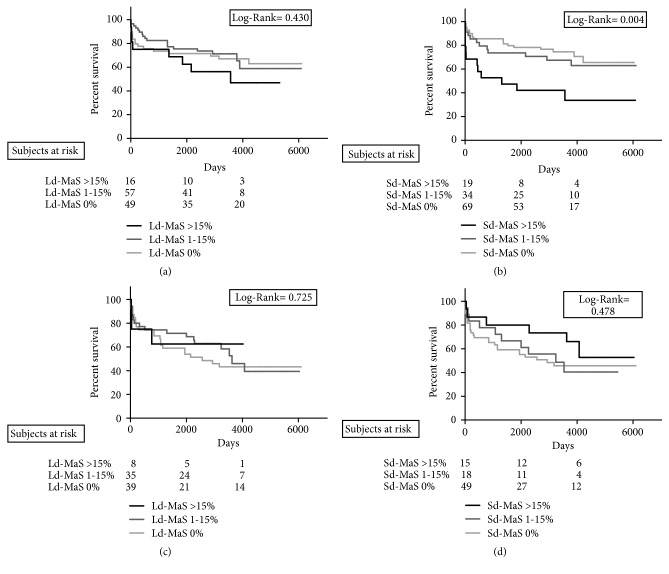
Cumulative overall graft survival rate according to graft large droplet (Ld-MaS; (a-c)) and small-droplet (Sd-MaS; (b-d)) macrovesicular steatosis distribution in recipients with HCV unrelated ((a), (b)) and related ((c), (d)) liver disease.

**Figure 4:**
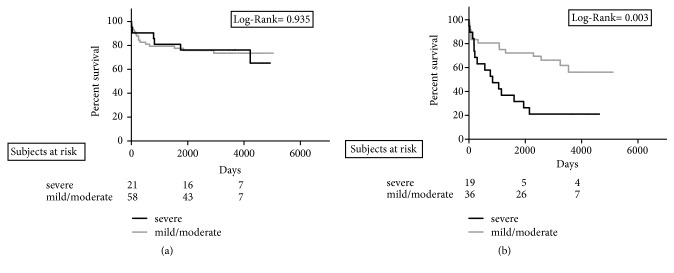
Cumulative overall graft survival rate according to graft histological ischemia/reperfusion injury severity in recipients with HCV unrelated (a) and HCV-related (b) liver disease.

Kaplan-Meier curves reporting the graft loss rates stratified for Ld-MaS >15% (Figure 3(a)) and Sd-MaS >15% (Figure 3(b)) showed that only this latter variable negatively influenced the survival results (Log Rank=0.004). As shown in Figure 4(a), IRI severity was not associated with graft loss.

**Figure 5:**
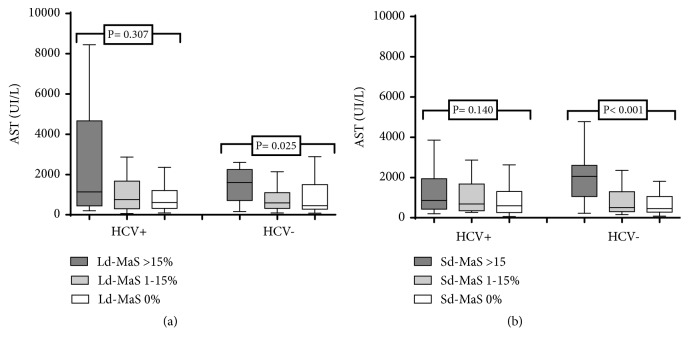
First three days after operative serum AST peak according to graft large droplet (Ld-MaS) and small-droplet (Sd-MaS) macrovesicular steatosis distribution in recipients with HCV unrelated (a) and related (b) liver disease.

Figure 5(a) reported that serum AST peaks observed during the first 3 post-LT days were significantly higher in HCV- patients receiving a graft with Sd-MaS >15% compared to patients with grafts with no Sd-MaS or <15% (*P*<0.001). Similar results were observed after using grafts with Ld-MaS >15% (*P*=0.025) (Figure 5(b)).

### 3.3. Variables Associated with Graft Survival in HCV-Positive Patients

The median follow-up was 7.1 years (range: 0.0-16.7). During follow-up, 32 grafts (39.0 %) were lost for liver-related causes. Specifically, we observed 19 recurrences of HCV-related cirrhosis, five delayed graft dysfunctions, two primary nonfunctions, two HCC recurrences, one hepatic artery thrombosis, one chronic rejection, one ischemic cholangitis, and one hepatic artery aneurysm. Ten (12.2%) grafts were lost due to liver-unrelated causes: five cerebrovascular accidents, one de novo malignancy, one sepsis, one acute myocardial infarction, one pulmonary embolism, and one intra-abdominal hemorrhage. One-, 3-, and 5-year graft survival rates were 75.6%, 67.1%, and 63.4%, respectively. As shown in Figure 1, six out of fifteen grafts with Sd-MaS >15% were lost. At univariable Cox regression analysis, length of donor intensive care unit (ICU) stay (*P*=0.015), graft cold (*P*<0.001) and warm ischemia (*P*=0.017) times, occurrence of IPGF (*P*<0.001) and EAD (*P*=0.049), and the severity of graft histopathological IRI (*P*=0.004) were significant risk factors for graft loss. Graft Ld-MaS and Sd-MaS, HCC, and other studied variables were not associated with overall graft survival (Table 2).

At multivariable Cox regression analyses (Table 4), graft cold ischemia time was the only significant (*P*<0.001) variable associated with graft loss in the pre-/intraoperative model. When also the postoperative variables were considered, the severity of graft IRI (P=0.002) and the occurrence of IPGF (*P*=0.009) were associated with graft loss.

No statistical differences were found in terms of survival rates when the cohort of HCV+ patients was stratified according to Ld-MaS and Sd-MaS values (Figures 3(c) and 3(d)). Severe IRI negatively influenced graft survival (Log Rank=0.003) (Figure 4(b)). In particular, among the 15 grafts with a severe IRI, 8 (53.3%) were lost due to HCV-related cirrhosis recurrence at a median post-LT time of 2.2 years (range: 0.5-5.9), while among the 14 grafts with a mild/moderate IRI, only 2 (14.3%) were lost due to HCV cirrhosis recurrence at 6.3 and 9.7 post-LT years, respectively.

In HCV+ patients, serum AST peaks observed during the first 3 postoperative days did not differ according to both Sd-MaS and Ld-MaS distribution (Figures 5(a) and 5(b)).

### 3.4. Donor Variables Associated with Graft Sd-MaS

Since the negative effects on postoperative aminotransferases and graft survival were observed in case of Sd-MaS >15%, we investigated the donor-specific factors associated with a Sd-MaS >15%. At univariable logistic regression analysis, risk factors for Sd-MaS >15% were a higher donor BMI (*P*=0.048), a shorter length of donor ICU stay (*P*=0.048), and a lower donor PaO_2_ (*P*=0.020) (Table 5). At multivariable binary logistic regression, a shorter length of donor ICU stay (*P*=0.023) and a lower donor PaO_2_ (*P*=0.019) were independent risk factors for Sd-MaS >15% (Table 5).

### 3.5. Graft ATP Content

**Figure 6:**
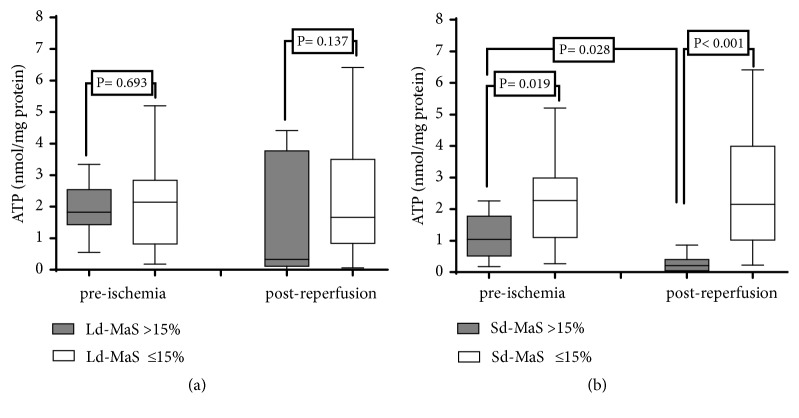
Graft ATP content at preischemia and postreperfusion according to graft large-droplet (Ld-MaS, (a)) and small-droplet (Sd-MaS, (b)).

A subanalysis was performed in a cohort of 42 grafts in which we measured preischemia and postreperfusion hepatic ATP content (Figure 6). Grafts with Sd-MaS >15% in the preischemia biopsy showed a significantly lower hepatic ATP content compared to grafts with lower rates of Sd-MaS (*P*=0.019). In addition, only grafts with Sd-MaS >15% in the preischemia biopsy significantly reduced ATP content in the postreperfusion biopsy when compared to preischemic results (*P*=0.028).

## 4. Discussion

In the present study we have investigated the impact on LT outcomes of donor liver steatosis evaluated using protocol preischemia biopsies and revised according to the Brunt classification. This classification identifies three different types of steatosis, namely, two subtypes of macrovesicular steatosis (Ld-MaS and Sd-MaS) and true microvesicular steatosis [11, 14]. This classification better distinguishes Sd-MaS from the true microvesicular steatosis with respect to the classically used models, improving reproducibility and avoiding the use of the term “microvesicular steatosis” interchangeably for the two different types of steatosis. Prior to the Brunt classification, the variable definition and interpretation of steatosis subtypes may explain the lack of consensus observed in many studies regarding their role with respect to post-LT outcomes [3, 5, 10]. In agreement with a recent report [13], we have found that the true microvesicular steatosis is virtually absent in our organ donor population. This is probably caused by the donor selection process, in which conditions associated with true microvesicular steatosis are typically excluded (i.e., hepatic encephalopathy and liver failure, Reye syndrome, drug toxicities, acute alcohol exposure, and acute fatty liver of pregnancy) [12, 14].

We have conducted separate analyses for graft survival in HCV+ and HCV- recipients. The main result of our study is that liver donor Sd-MaS is an independent risk factor for graft loss in HCV- patients, but not in HCV+ ones. In particular, graft Sd-MaS was associated with graft loss when considered as either a continuous variable or categorized using a cut-off of >15%.

Although the accuracy of frozen liver sections is debated for steatosis assessment [14], our results may suggest that the role of biopsy is underutilized in the graft selection process, mainly in case of donors with high risk of steatosis or previously documented steatosis at ultrasound. In fact, in contrast to Ld-MaS, the presence and the quantity of Sd-MaS are poorly evaluable by the surgeon when the graft steatosis is grossly estimated during the organ procurement [13].

To date, only one recently published study by Choi et al. has analyzed the impact of Sd-MaS and Ld-MaS on LT outcomes, finding an association of Sd-MaS with acute and chronic rejection, but not with graft survival [15]. The discrepant results on graft survival between our present and Choi's study could be due to several reasons. First, in Choi's study the liver donor biopsy was not performed per protocol in all cases, as in our study, but only when the surgeons suspected the presence of steatosis. This introduces selection biases, like missing cases with significant histologically detectable, but poorly suspected at gross inspection, Sd-MaS, and excluding from analyses many livers with no steatosis [13]. Furthermore, in Choi's study no separate analysis was performed according to HCV recipient status [15]. This has potentially masked the effect of Sd-MaS on graft survival, since in our present study HCV+ patients receiving a graft with Sd-MaS >15% did not show worse survival rates.

With regard to the mechanisms through which allograft with relevant Sd-MaS have a poor outcome in recipients with HCV- liver disease, we found that these grafts (a) had low ATP content in the preischemia biopsy, suffering a further significant reduction of ATP after reperfusion; (b) were associated with low donor PaO_2_ and short length of ICU stay; and (c) when transplanted to HCV- recipients, showed a higher early postoperative serum AST peak, compared to the other grafts. Thus, we hypothesize that donors with relevant Sd-MaS have a preexisting impaired mitochondrial function with low baseline ATP content, failure to recover ATP levels after reoxygenation and increased susceptibility to ischemia-reperfusion injury [23–26]. The mitochondrial damage and reduced ATP synthesis are further worsened in the case of hypoxia. Hyperoxia protects from these events, as have been shown in explanted rat livers by others and in human donors by us [27, 28]. As ICU stay and reduced caloric intake prolong, Sd-MaS is then reduced by lipophagy activation, in keeping with two previous observations: (a) the upregulation of lysosomal lipase, a lipid droplet catabolizing enzyme, under starving conditions of primary hepatocytes and (b) the pronounced reduction of the classically termed “microvesicular steatosis” shown in steatotic livers of potential living donors for LT submitted to low-calorie diet [29].

In our present study we did not find a negative impact of Sd-MaS on graft survival and early postoperative AST peak in HCV+ patients. Although we do not have a clear explanation for this latter observation, we should underline the fact that HCV is known to strictly interact with lipid droplets into the hepatocytes, redirecting autophagy by inducing lipid-selective autophagy [16, 30]. In accordance, it has been previously reported a strong negative correlation between the level of autophagy and “microvesicular” steatosis in HCV-infected patients, but not in patients with nonalcoholic liver disease [31]. As a consequence, it should be speculated that HCV modulates autophagy in a way that reduces hepatocellular damage due to the presence of Sd-MaS [32].

With regard to predictors of graft loss in our HCV+ patients, we found that, as previously reported, severe IRI was associated with cirrhosis due to HCV recurrence [33].

The definition of Ld-MaS in the present study is concordant with the term macrosteatosis/ macrovesicular steatosis used in the literature; according to several studies, when macrosteatosis occurs in more than 60% of hepatocytes, poor outcomes are observed [2–4, 13]. However, in agreement with Choi's study, we did not find any association between Ld-MaS and LT transplant outcomes [15]. This is probably because of two reasons: (a) the maximum Ld-MaS value in our study was 40%, since we discarded grafts with a classically termed macrovesicular steatosis at frozen sections exceeding this value; (b) as it is the practice of many transplant centers, we allocated grafts with a high “macrovesicular steatosis” to patients with low MELD scores (data not shown).

The observed result that donor Anti-HBc positivity was connected with poor graft survivals in HCV- patients is in line with previous studies [34]. However, the suggestion that the Anti-HBc positivity may be a surrogate marker of low graft quality is only a hypothetical, the possible underlying mechanisms for this phenomenon still being unclear.

There are some limitations of our study that should be addressed. First of all, this is a monocentric study needing an external validation of our results. The study was performed in a long time frame. However, this possible bias was corrected adding the period of transplant as a covariate in our analyses. Although we performed only one liver biopsy of the left hepatic lobe to assess preischemia steatosis, previous studies have shown minimal steatosis variability between left and right lobe sampling [35]. Lastly, despite the division in two populations (HCV-RNA negative and positive) reducing the statistical power, this splitting was necessary in order to avoid the possible confounding role of HCV and to give to the study a forward-looking perspective because of the progressive reduction of LT candidates with HCV-RNA positivity.

In conclusion, using protocol preischemia liver graft biopsies, we observed that the presence of Sd-MaS >15% is associated with lower graft ATP content, severe early hepatocellular damage, and reduced graft survival in HCV-negative patients. These data may play an important role in modifying the organ allocation process, especially nowadays with the spreading of NASH and the reduction of HCV-RNA positive recipients thank to DAAs, but need to be validated in other studies.

## Figures and Tables

**Table 1:** Recipient, donor, graft, intraoperative, and early postoperative characteristics of the entire study population and according to recipient etiology of liver disease (HCV negative versus HCV positive).

		All patients (n= 204)	HCV positive (n= 82)	HCV negative (n= 122)	*P* value HCV positive vs HCV negative
RECIPIENT	Age (years)	56.00 (49.14-61.00)	57.00 (49.00-61.00)	55.50 (49.75-61.25)	0.510
	Gender (female)	49 (24.0)	22 (26.8)	27 (22.1)	0.441
	MELD score	15.20 (12.26-18.65)	14.73 (12.46-18.85)	15.72 (11.78-18.15)	0.549
	BMI (kg/m^2^)	25.46 (23.28-28.47)	26.23 (23.91-28.64)	25.06 (23.04-28.34)	0.184
	HCC (yes vs no)	78 (38.2)	32 (39.0)	46 (37.7)	0.849

DONOR	Age (years)	50.50 (33.25-64.00)	48.00 (31.00-65.00)	51.00(34.00-64.00)	0.703
	Gender (female)	84 (41.2)	32 (39.0)	52 (42.6)	0.609
	BMI (kg/m^2^)	24.83 (23.44-27.06)	24.69 (23.44-26.15)	25.39 (23.61-27.34)	0.131
	Cause of death (non trauma vs trauma)	132 (65.7)	50 (61)	82 (68.9)	0.244
	ALT (IU/L)	33.00 (18.00-58.50)	33.00 (18.00-59.00)	32.50 (17.75-58.50)	0.562
	AST (IU/L)	37.50 (25.00-70.50)	45.50 (24.00-83.00)	36.00 (25.00-58.50)	0.174
	Sodium (mEq/L)	149.00 (142.25-157.00)	150.00 (142.00-157.00)	149.00 (144.00-157.00)	0.804
	Hemoglobin (gr/dL)	10.50 (9.30-12.20)	10.40 (9.00-11.90)	10.70 (9.50-12.30)	0.440
	PaO_2_ (mmHg)	150.50 (102.93-202.08)	148.90 (111.50-217.50)	151.00 (98.00-198.00)	0.776
	Anti-HBc status (pos vs neg)	18 (8.8)	3 (3.7)	15 (12.3)	**0.043**
	Norepinephrine (yes vs no)	98 (49.0)	33 (40.7)	65 (54.6)	0.054
	ICU stay (days)	3.00 (2.00-7.00)	3.00 (2.00-6.00)	4.00 (2.00-8.00)	0.113

GRAFT	Sd-MaS categorical, n (%):				
	0%	118 (57.8)	49 (59.8)	69 (56.6)	0.613
	1-15%	52 (25.5)	18 (22.0)	34 (27.9)	
	>15%	34 (16.7)	15 (18.3)	19 (15.6)	
	Sd-MaS, continuous variable (% of hepatocytes)	0.00 (0.00-5.00)	0.00 (0.00-10.00)	0.00 (0.00-5.00)	0.969
	Ld-MaS categorical, n (%):				
	0% (reference)	88 (43.1)	39 (47.6)	49 (40.2)	0.530
	1-15%	92 (45.1)	35 (42.7)	57 (46.7)	
	>15%	24 (11.8)	8 (9.8)	16 (13.1)	
	Ld-MaS, continuous variable (% of hepatocytes)	2.00 (0.00-9.00)	1.00 (0.00-5.00)	2.00 (0.00-10.00)	0.245
	Cold ischemia time (minutes)	361.00 (280.75-415.00)	357.50 (270.00-421.25)	362.50 (298.25-410.00)	0.606
	Warm ischemia time (minutes)	60.00 (47.50-77.75)	60.00 (45.75-87.75)	60.00 (48.50-75.00)	0.316
	IRI score, categorical^§^ (severe vs mild/moderate)	40 (29.9)	19 (34.5)	21 (26.6)	0.322
	IPGF (yes vs no)	37 (18.3)	15 (18.5)	22 (18.2)	0.952
	EAD (yes vs no)	112 (54.9)	48 (58.5)	64 (52.5)	0.392
	Transplant year	6.00 (3.00-8.75)	5.00 (3.00-7.00)	6.00 (3.00-9.00)	0.105

MELD, model for end-stage liver disease score; HCC, hepatocellular carcinoma; PaO_2_, partial pressure of oxygen in arterial blood; ICU, intensive care unit; Sd-MaS, small droplet macrovesicular steatosis; Ld-MaS, large droplet macrovesicular steatosis; IRI, histological ischemia/reperfusion injury; IPGF, initial poor graft function; EAD, early allograft dysfunction. Continuous variable is expressed as median (25th-75th percentile); the differences between groups were evaluated by Mann-Whitney *U* test or T test according to the variable normality. Categorical variables were expressed as count (percentages) and compared by the chi-square or Fisher's exact test.

§Available in only 55 and 79 recipients with HCV positive and negative liver disease, respectively.

**Table 2:** Univariable Cox regression analyses for overall graft loss according to recipient etiology of liver disease (HCV negative versus HCV positive).

		*HCV positive*			*HCV negative*		
		*HR*	(95% CI)	*P*	*HR*	(95% CI)	*P*
RECIPIENT	Age (years)	0.988	0.951-1.026	0.517	0.998	0.973-1.024	0.892
	Gender (female vs male)	1.794	0.954-3.376	0.070	1.052	0.518-2.135	0.888
	MELD score	0.993	0.928-1.063	0.845	1.050	0.993-1.110	0.089
	BMI (kg/m^2^)	1.065	0.967-1.173	0.204	1.001	0.929-1.079	0.979
	HCC (yes vs no)	1.139	0.616-2.103	0.678	1.338	0.734-2.439	0.342

DONOR	Age (years)	1.009	0.993-1.026	0.284	1.014	0.996-1.032	0.119
	Gender (female vs male)	0.983	0.530-1.821	0.955	1.326	0.729-2.413	0.355
	BMI (kg/m^2^)	1.034	0.919-1.162	0.580	0.954	0.869-1.046	0.314
	Cause of death (non trauma vs trauma)	1.014	0.544-1.891	0.965	0.867	0.456-1.650	0.663
	ALT (IU/L)	1.000	0.995-1.005	0.948	0.998	0.992-1.004	0.590
	AST (IU/L)	0.996	0.990-1.002	0.148	0.999	0.993-1.005	0.797
	Sodium (mEq/L)	1.008	0.979-1.038	0.594	0.995	0.964-1.027	0.742
	Hemoglobin (gr/dL)	0.993	0.868-1.137	0.923	1.049	0.916-1.201	0.491
	PaO_2_ (mmHg)	1.000	0.996-1.004	0.872	0.999	0.995-1.002	0.557
	Anti-HBc status (pos vs neg)	1.424	0.344-5.902	0.626	3.190	1.565-6.501	**0.001**
	Norepinephrine (yes vs no)	1.512	0.813-2.811	0.191	0.588	0.315-1.0.97	0.095
	ICU stay (days)	1.121	1.022-1.229	**0.015**	0.555	0.954-1.091	0.555

GRAFT	Sd-MaS categorical, n (%):						
	0%						
	1-15%	0.939	0.453-1.948	0.866	1.284	0.622-2.647	0.499
	>15%	0.581	0.239-1.415	0.232	3.146	1.525-6.489	**0.002**
	Sd-MaS, continuous variable (% of hepatocytes)	0.983	0.958-1.010	0.212	1.036	1.018-1.055	**<0.001**
	Ld-MaS categorical, n (%):						
	0% (reference)						
	1-15%	0.841	0.445-1.586	0.592	0.694	0.360-1.337	0.275
	>15%	0.650	0.194-2.177	0.484	1.430	0.654-3.129	0.371
	Ld-MaS, continuous variable (% of hepatocytes)	0.983	0.944-1.024	0.406	1.016	0.992-1.040	0.193
	Cold ischemia time (minutes)	1.007	1.003-1.010	**<0.001**	1.001	0.998-1.005	0.364
	Warm ischemia time (minutes)	1.015	1.003-1.027	**0.017**	0.997	0.982-1.012	0.718
	IRI score, categorical^§^ (severe vs mild/moderate)	2.932	1.399-6.145	**0.004**	0.961	0.365-2.527	0.935
	IPGF (yes vs no)	3.340	1.694-6.584	**<0.001**	2.152	1.100-4.212	**0.025**
	EAD (yes vs no)	1.839	1.004-3.371	**0.049**	2.346	1.252-4.396	**0.008**
	Transplant year	0.954	0.854-1.067	0.411	0.900	0.816-0.993	**0.036**

MELD, model for end-stage liver disease score; HCC, hepatocellular carcinoma; BMI, body mass index; PaO_2_, partial pressure of oxygen in arterial blood; ICU, intensive care unit; Sd-MaS, small droplet macrovesicular steatosis; Ld-MaS, large droplet macrovesicular steatosis; IRI, histological ischemia/reperfusion injury; IPGF, initial poor graft function; EAD, early allograft dysfunction.

§Available in only 55 and 79 recipients with HCV positive and negative liver disease, respectively.

**Table 3:** Multivariable Cox regression models for overall graft loss in recipients with HCV negative liver disease.

	*Pre-/intra-operative model*			*Early post-operative model*		
	*HR*	(95% CI)	*P*	*HR*	(95% CI)	*P*
Donor anti-HBc serum status (positive versus negative)	3.303	1.601-6.814	**0.001**	2.855	1.329-6.134	**0.007**
Sd-MaS categorical, n (%): 0% (reference)						
1-15%	1.223	0.587-2.547	0.591	1.357	0.641-2.871	0.425
>15%	2.891	1.312-6.369	**0.008**	2.623	1.169-5.888	**0.019**
IPGF (yes vs no)				1.094	0.494-2.426	0.824
EAD (yes vs no)				1.849	0.891-3.838	0.099
Transplant year	0.947	0.851-1.053	0.315	0.969	0.863-1.087	0.589

Sd-MaS, small droplet macrovesicular steatosis; IPGF, initial poor graft function; EAD, early allograft dysfunction.

**Table 4:** Multivariable Cox regression models for overall graft loss in recipients with HCV positive liver disease.

	*Pre-/intra-operative model*			*Early post-operative model*		
	*HR*	(95% CI)	*P*	*HR*	(95% CI)	*P*
Donor ICU stay (days)	1.078	0.983-1.182	0.112	0.967	0.855-1.094	0.596
Graft cold ischemia time (minutes)	1.006	1.003-1.010	**<0.001**	1.004	1.000-1.008	0.050
Graft warm ischemia time (minutes)	1.011	0.999-1.024	0.073	1.017	0.998-1.035	0.077
IRI score, categorical (severe vs mild/moderate)^§^				4.485	1.755-11.459	**0.002**
IPGF (yes vs no)				5.074	1.499-17.170	**0.009**
EAD (yes vs no)				0.921	0.370-2.292	0.860

ICU, intensive care unit; IRI, histological ischemia/reperfusion injury; IPGF, initial poor graft function; EAD, early allograft dysfunction.

§Available in only 55 patients.

**Table 5:** Univariable and multivariable binary logistic regression analysis of donor variables associated with graft Sd-MaS.

	Sd-MaS≤ 15% (n=170)	Sd-MaS >15% (n=34)	P	OR	(95% CI)	P
Age (years)	50.00 (33.00-65.00)	52.00 (39.00-61.25)	0.790			
Gender (female versus male)	75 (44.1)	9 (26.50)	0.056			
BMI (kg/m2)	24.69 (23.44-26.36)	26.12 (24.05-27.71)	**0.048**	1.124	0.994-1.271	0.063
Cause of death (non trauma vs trauma)	114 (67.9)	18 (54.50)	0.141			
ALT (IU/L)	33.00 (18.00-58.00)	33.00 (21.00-63.00)	0.476			
AST (IU/L)	36.00 (25.00-69.00)	49.00 (28.00-78.00)	0.146			
Sodium (mEq/L)	149.00 (142.00-154.00)	150.00 (142.50-163.50)	0.180			
Hemoglobin (g/dL)	10.40 (9.05-12.10)	11.00 (9.80-13.20)	0.075			
PaO_2_ (mmHg)	154.80 (107.00-219.00)	122.00 (89.50-168.50)	**0.020**	0.993	0.986-0.999	**0.019**
Anti-HBc status (pos vs neg)	13 (7.6)	5 (14.7)	0.185			
Norepinephrine (yes vs no)	86 (51.5)	12 (36.4)	0.112			
ICU stay (days)	4.00 (2.00-7.00)	3.00 (2.00-4.00)	**0.048**	0.851	0.740-0.978	**0.023**

Data are reported as means and standard deviations for normally distributed or medians (25th-75th percentile) for nonnormally distributed ones. Absolute and relative frequencies are reported for categorical ones.

Differences between groups were tested with Mann-Whitney *U* test for continuous variables and with chi-square test or Fisher exact probability test for categorical ones.

PaO_2_, partial pressure of oxygen in arterial blood; ICU, intensive care unit.

## Data Availability

The clinical data used to support the findings of this study are included within the article in anonymous form in order to protect patient privacy as request by the local ethical committee.

## Abbreviations

DAAs: Direct-Acting Antivirals
EAD: Early allograft dysfunction
HCC: Hepatocellular carcinoma
IPGF: Initial poor graft function
IRI: Ischemia/reperfusion injury
Ld-MaS: Large droplet macrovesicular
LT: Liver transplantation
MiS: True microvesicular
PaO_2_: Partial pressure of oxygen
Sd-MaS: Small-droplet macrovesicular.

## References

[1] Selzner M., Clavien P.-A. (2001). Fatty liver in liver transplantation and surgery. *Seminars in Liver Disease*.

[2] Durand F., Renz J. F., Alkofer B. (2008). Report of the Paris consensus meeting on expanded criteria donors in liver transplantation. *Liver Transplantation*.

[3] Spitzer A. L., Lao O. B., Dick A. A. S. (2010). The biopsied donor liver: incorporating macrosteatosis into high-risk donor assessment. *Liver Transplantation*.

[4] Chu M. J., Dare A. J., Phillips A. R., Bartlett A. S. (2015). Donor hepatic steatosis and outcome after liver transplantation: a systematic review. *Journal of Gastrointestinal Surgery*.

[5] Fishbein T. M., Fiel M. I., Emre S. (1997). Use of livers with microvesicular fat safely expands the donor pool. *Transplantation*.

[6] Ureña M. A. G., Ruiz-Delgado F. C., González E. M. (1998). Assessing risk of the use of livers with macro and microsteatosis in a liver transplant program. *Transplantation Proceedings*.

[7] Cieślak B., Lewandowski Z., Urban M., Ziarkiewicz-Wróblewska B., Krawczyk M. (2009). Microvesicular liver graft steatosis as a risk factor of initial poor function in relation to suboptimal donor parameters. *Transplantation Proceedings*.

[8] Suzuki S., Toledo-Pereyra L. H., Rodriguez F. J., Cejalvo D. (1993). Neutrophil infiltration as an important factor in liver ischemia and reperfusion injury. Modulating effects of FK506 and cyclosporine. *Transplantation*.

[9] Noujaim H. M., de Ville de Goyet J., Montero E. F. (2009). Expanding postmortem donor pool using steatotic liver grafts: a new look. *Transplantation*.

[10] Yoong K. F., Gunson B. K., Neil D. A. H. (1999). Impact of donor liver microvesicular steatosis on the outcome of liver retransplantation. *Transplantation Proceedings*.

[11] Brunt E. M. (2007). Pathology of fatty liver disease. *Modern Pathology*.

[12] Ramachandran R., Kakar S. (2009). Histological patterns in drug-induced liver disease. *Journal of Clinical Pathology*.

[13] Yersiz H., Lee C., Kaldas F. M. (2013). Assessment of hepatic steatosis by transplant surgeon and expert pathologist: A prospective, double-blind evaluation of 201 donor livers. *Liver Transplantation*.

[14] Brunt E. M. (2013). Surgical assessment of significant steatosis in donor livers: The beginning of the end for frozen-section analysis?. *Liver Transplantation*.

[15] Choi W.-T., Jen K.-Y., Wang D., Tavakol M., Roberts J. P., Gill R. M. (2017). Donor liver small droplet macrovesicular steatosis is associated with increased risk for recipient allograft rejection. *The American Journal of Surgical Pathology*.

[16] Modaresi Esfeh J., Ansari-Gilani K. (2016). Steatosis and hepatitis C. *Gastroenterology Report*.

[17] Leandro G., Mangia A., Hui J. (2006). Relationship between steatosis, inflammation, and fibrosis in chronic hepatitis C: a meta-analysis of individual patient data. *Gastroenterology*.

[18] Briceño J., Ciria R., Pleguezuelo M. (2009). Impact of donor graft steatosis on overall outcome and viral recurrence after liver transplantation for hepatitis C virus cirrhosis. *Liver Transplantation*.

[19] Salizzoni M., Franchello A., Zamboni F. (2003). Marginal grafts: finding the correct treatment for fatty livers. *Transplant International*.

[20] Nanashima A., Pillay P., Verran D. J. (2002). Analysis of initial poor graft function after orthotopic liver transplantation: experience of an australian single liver transplantation center. *Transplantation Proceedings*.

[21] Olthoff K. M., Kulik L., Samstein B. (2010). Validation of a current definition of early allograft dysfunction in liver transplant recipients and analysis of risk factors. *Liver Transplantation*.

[22] Burra P., Germani G., Adam R. (2013). Liver transplantation for HBV-related cirrhosis in Europe: An ELTR study on evolution and outcomes. *Journal of Hepatology*.

[23] Fromenty B., Pessayre D. (1997). Impaired mitochondrial function in microvesicular steatosis: Effects of drugs, ethanol, hormones and cytokines. *Journal of Hepatology*.

[24] Fromenty B., Berson A., Pessayre D. (1997). Microvesicular steatosis and steatohepatitis: Role of mitochondrial dysfunction and lipid peroxidation. *Journal of Hepatology*.

[25] Evans Z. P., Palanisamy A. P., Sutter A. G. (2012). Mitochondrial uncoupling protein-2 deficiency protects steatotic mouse hepatocytes from hypoxia/reoxygenation. *American Journal of Physiology-Gastrointestinal and Liver Physiology*.

[26] Hashani M., Witzel H. R., Pawella L. M. (2018). Widespread expression of perilipin 5 in normal human tissues and in diseases is restricted to distinct lipid droplet subpopulations. *Cell and Tissue Research*.

[27] Sgarbi G., Giannone F., Casalena G. A. (2011). Hyperoxia fully protects mitochondria of explanted livers. *Journal of Bioenergetics and Biomembranes*.

[28] Corradini S. G., Elisei W., De Marco R. (2005). Preharvest donor hyperoxia predicts good early graft function and longer graft survival after liver transplantation. *Liver Transplantation*.

[29] Hwang S., Lee S.-G., Jang S.-J. (2004). The effect of donor weight reduction on hepatic steatosis for living donor liver transplantation. *Liver Transplantation*.

[30] Hara Y., Yanatori I., Ikeda M. (2014). Hepatitis C virus core protein suppresses mitophagy by interacting with parkin in the context of mitochondrial depolarization. *The American Journal of Pathology*.

[31] Vescovo T., Romagnoli A., Perdomo A. B. (2012). Autophagy protects cells from hcv-induced defects in lipid metabolism. *Gastroenterology*.

[32] Cursio R., Colosetti P., Gugenheim J. (2015). Autophagy and liver ischemia-reperfusion injury. *BioMed Research International*.

[33] Watt K. D. S., Lyden E. R., Gulizia J. M., McCashland T. M. (2006). Recurrent hepatitis C posttransplant: Early preservation injury may predict poor outcome. *Liver Transplantation*.

[34] Angelico M., Nardi A., Marianelli T. (2013). Hepatitis B-core antibody positive donors in liver transplantation and their impact on graft survival: Evidence from the Liver Match cohort study. *Journal of Hepatology*.

[35] Larson S. P., Bowers S. P., Palekar N. A., Ward J. A., Pulcini J. P., Harrison S. A. (2007). Histopathologic variability between the right and left lobes of the liver in morbidly obese patients undergoing roux-en-Y bypass. *Clinical Gastroenterology and Hepatology*.

